# Adrenal Vein Sampling: Does the Location of the Non-adrenal Venous Sample Matter?

**DOI:** 10.1007/s00270-023-03647-z

**Published:** 2024-01-11

**Authors:** Florian Wernig, Aleksandra Dunin-Borkowska, Angelos Frisiras, Bernard Khoo, Jeannie Todd, Aimee Di Marco, F. Fausto Palazzo, Sophie C. Barnes, Tricia M. Tan, Karim Meeran, Ali Alsafi

**Affiliations:** 1 Department of Endocrinology, Imperial College Healthcare NHS Trust, London, UK; 2 Imaging Department, Hammersmith Hospital, Imperial College Healthcare NHS Trust, Du Cane Road, London, W12 0HS UK; 3 Department of Endocrinology, Division of Medicine, Royal Free Campus, University College London, London, UK; 4 Department of Endocrine Surgery, Hammersmith Hospital, Imperial College Healthcare NHS Trust, London, UK; 5 Department of Surgery & Cancer, Imperial College, London, UK; 6 Department of Clinical Biochemistry, North-West London Pathology, London, UK; 7 Division of Diabetes, Endocrinology and Metabolism, Imperial College London, London, UK

**Keywords:** Primary hyperaldosteronism, Primary aldosteronism, Hypertension, Conn’s, Adrenal, Adrenal vein sampling

## Abstract

**Purpose:**

Adrenal vein sampling (AVS) is used to lateralise and differentiate unilateral from bilateral aldosterone production in primary aldosteronism. The adrenal venous samples are standardised to a peripheral or low inferior vena cava (IVC) sample and compared. It is unknown whether the location of the non-adrenal sample affects the results. This study compares AVS results standardised to the low IVC and right external iliac vein (REIV).

**Methods:**

Patients who underwent AVS between March 2021 and May 2023 were included. All procedures were undertaken by a single operator (AA). Demographic data and AVS results were collected from patients’ electronic records. Catheterisation success and lateralisation were assessed using both low IVC and REIV samples. Equivalence test was used to compare the cortisol and aldosterone levels.

**Results:**

Eighty-one patients, (M: F = 38:43), aged between 29 and 74 were included. Bilateral successful adrenal vein cannulation was achieved in 79/81 (97.5%) cases. The mean cortisol levels from the REIV were statistically equivalent although there was a small and not biologically significant difference from the low IVC (respective geometric means 183 nmol/l vs. 185 nmol/l, *p* = 0.015). This small difference in cortisol may be due to accessory adrenal venous drainage into the IVC. The aldosterone and aldosterone/cortisol ratios were statistically equivalent. There was no discordance in selectivity or lateralisation when the IVC or REIV measurements were used.

**Conclusion:**

The IVC and REIV samples may be used interchangeably during AVS.

**Graphical Abstract:**

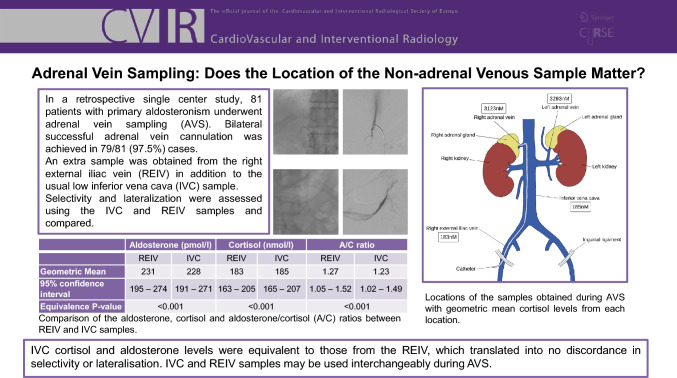

## Introduction

Primary aldosteronism is characterised by the overproduction of aldosterone in the absence of renin stimulation [1]. It is the most common cause of secondary hypertension and can either be caused by a unilateral aldosterone-producing adrenal adenoma or bilateral disease [2]. In patients diagnosed biochemically with primary aldosteronism who are willing to undergo surgery, it is important to determine whether the excess aldosterone production is unilateral or bilateral to guide appropriate management [3]. Unilateral disease can be successfully treated with adrenalectomy, whilst pharmacological treatment is indicated in bilateral disease. The gold-standard test for determining laterality is adrenal vein sampling (AVS) [1, 4].

Whilst AVS is the most reliable method of identifying subtypes of primary aldosteronism, it is a technically challenging procedure with a significant reported failure rate, most often due to failure of catheterisation of the right adrenal vein [5–7]. It is important to note, however, that much lower failure rates are achieved at high-volume centres [4].

Cortisol levels are used to determine successful catheterisation. Catheterisation is said to be successful when the cortisol levels from the adrenal veins are at least twice those of samples obtained from the low inferior vena cava (IVC) or a peripheral sample when AVS is done without ACTH stimulation [5, 8]. Different cut-offs exist for ACTH-stimulated AVS [5–12]. It is unclear whether the location of the non-adrenal venous sample could influence the interpretation of AVS. To answer this question, we exploited a change in our AVS protocol in 2021, which stipulated the collection of a right external iliac vein (REIV) sample in addition to the usual IVC sample. The change in the protocol was due to several cases of undetectable aldosterone levels in the IVC samples in the preceding years. This study compares AVS results standardised to the low IVC and REIV.

## Materials and Methods

Patients who underwent AVS between March 2021 and May 2023 were identified using the Radiology Information System. Their demographic data, alongside the AVS results and procedure-related complications, were collected from the patients’ electronic records.

### Adrenal Vein Sampling Technique

AVS technique has been extensively described previously [13]. Briefly, all procedures were performed as a day case by a single interventional radiologist (AA). Sequential, non-stimulated venous samples were obtained from both adrenal veins, low IVC (Approximately 5 cm below the lowest renal vein) and REIV via a right femoral venous approach using a 4Fr Glidecath^©^ hydrophilic coated Cobra catheter (Terumo, Tokyo Japan) or a Sidewinder II catheter (Cardinal Health, Santa Clara, CA, USA). Two side-holes were punched in the catheters’ sagittal plane 1 to 2 mm away from the tip. Venography was performed to verify correct catheter position. Each sample bottle was numbered, and the numbers were transcribed onto a diagram of normal venous anatomy [13–15].

### Adrenal Vein Sampling Catheterisation Success

To confirm correct adrenal vein cannulation, the adrenal vein sample should have a cortisol level that is twice as high as that of the sample taken from the low IVC or REIV. The ratio of the cortisol concentration in the adrenal vein to that in the peripheral vein is known as the selectivity index (SI) [16]. If venography was discordant with the cortisol results or an aldosterone/cortisol co-secreting adenoma was suspected, metanephrine levels were used to confirm catheter placement. In these cases, an adrenal vein to IVC or REIV metanephrine ratio of > 12 confirmed correct catheter placement [9–12, 17].

### Lateralisation

Unilateral disease was defined as aldosterone/cortisol (A/C) ratio of the dominant adrenal vein being greater than two times the A/C of the contralateral adrenal vein (i.e. Lateralisation Index (LI) = A/C_Dominant_/A/C_Contralateral_ > 2). This is in addition to suppression of the contralateral adrenal vein with an A/C ratio below half of that of the IVC or REIV (i.e. Contralateral suppression index (CSI) = A/C_Contralateral_/A/C_IVC or REIV_ ≤ 0.5).

Cortisol and aldosterone levels from the low IVC and REIV samples were compared. Catheterisation success and lateralisation were assessed using both IVC and REIV samples and compared.

### Analytical Methods

Serum samples were analysed for cortisol using the Abbott Alinity chemiluminescent microparticle immunoassay for cortisol with an analytical range 28–1650 nmol/l, precision, expressed as percentage coefficient of variation, of 7.2% at 100 nmol/l and < 4% at higher concentrations (Abbott Laboratories, Maidenhead, UK). Aldosterone analysis was by an in-house method for quantitation by liquid chromatography tandem mass spectrometry following solid phase extraction with analytical range 60–5500 pmol/l, precision ≤ 6.6% at all concentrations. Samples above the analytical range were analysed following dilution with appropriate diluents.

### Statistical Analysis

Stata 15.1 (STATACorp LLC) and the *tost* programme for tests of equivalence (https://www.alexisdinno.com/stata/tost.html) were used for statistical analysis. Values below the lowest limit of quantification were imputed as the lowest limit of quantification. Kernel density plots confirmed that the distribution of values was nonparametric and right-skewed, so a log-transformation was used for analysis. Geometric means and 95% confidence intervals are presented. A two-sample paired mean-equivalence *t*-test was used to compare the log-transformed IVC and REIV hormone levels. We specified equivalence levels of 32 nmol/l for cortisol and 100 pmol/l for aldosterone, based on the intra-individual biological variation of each hormone (https://www.westgard.com/biodatabase1.htm). We also specified 0.5 as the equivalence level for the A/C based on clinical experience.

## Results

Eighty-one patients underwent AVS for primary aldosteronism over the study period. The median age was 50 years [29–74]. Forty-three (53%) participants were female. There were no procedure-related complications. Three patients had their AVS repeated; two were due to an unsuccessful first attempt at adrenal vein catheterisation, and one was due to apparent bilateral suppression of aldosterone production. There were two cases of unsuccessful adrenal vein catheterisation: one left adrenal vein and one right adrenal vein. Overall, 79/81 (97.5%) of the AVS procedures were successful.

**Fig. 1:**
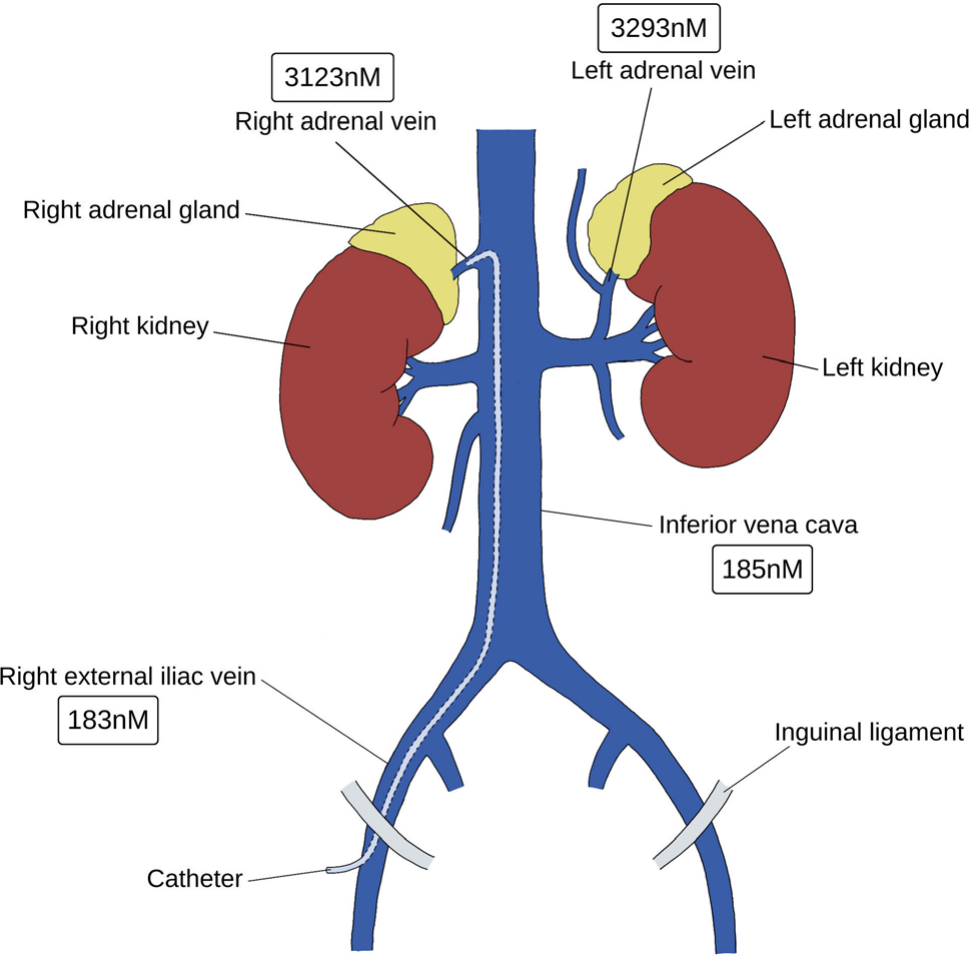
Illustration of the locations of the samples obtained during adrenal vein sampling with geometric mean cortisol levels from each location

The geometric mean cortisol levels from the REIV were statistically significantly lower than those from the low IVC (183 nmol/l vs. 185 nmol/l, *p* = 0.015) (Fig. 1). Equivalence testing, taking 32 nmol/l as the minimal significant difference, suggests that these are equivalent, in other words, even though the means were statistically significantly different, the difference was considered trivial and not biologically significant. The geometric mean aldosterone levels from the low IVC and REIV samples were not significantly different from each other and were considered equivalent on testing with a 100 pmol/l minimal significant difference Table 1. Low aldosterone levels (< 100 pmol/l) were recorded in 8/81 cases (REIV 92 pmol/l, vs. IVC 86 pmol/l) and undetectable (< 60 pmol/l) in 6 cases. There was no significant difference between the geometric mean A/C ratios between the samples obtained from the IVC and REIV, and these were considered equivalent on testing, with a minimum significant difference of 0.5. This translated into no discordance in lateralisation when the results were standardised to the IVC or REIV.

**Table 1:** Comparison of the aldosterone, cortisol and A/C ratios between REIV and IVC samples

	Aldosterone (pmol/l)		Cortisol (nmol/l)		A/C ratio	
	REIV	IVC	REIV	IVC	REIV	IVC
Min	< 60	< 60	55	56	0.3	0.4
Max	1250	1360	562	558	8.6	6.3
Geometric mean	231	228	183	185	1.27	1.23
95% confidence interval	195–274	191–271	163–205	165–207	1.05–1.52	1.02–1.49
Paired *t* test *p* value	0.292		0.015		0.093	
Equivalence *p* value	< 0.001		< 0.001		< 0.001	

Range, geometric means and 95% confidence intervals are presented. *p* values were calculated for a paired *t*-test of the log-transformed data and for an equivalence test (where *p* < 0.05 is considered equivalent)

## Discussion

This study’s findings demonstrate that selectivity and lateralisation were concordant when the IVC or REIV samples were used as surrogates for a peripheral venous sample during AVS. Whilst cortisol levels were significantly lower by a geometric mean difference of 1 nmol/l in the REIV samples compared with those from the low IVC, this was considered a trivial difference and resulted in no difference in lateralisation or selectivity. Some of the differences, however, may be attributable to variant adrenal venous anatomy [18, 19]. One or more inferior emissary veins arise from the surface of the adrenal glands and may communicate with the renal veins, intercostal veins, phrenic veins, or IVC [14, 20]. This, in turn, may result in some adrenal venous effluent draining more inferiorly, thereby resulting in slightly higher adrenal hormone levels in the low IVC compared to blood returning from the lower limbs (external iliac veins). Compression of the left renal vein by the superior mesenteric artery can result in the enlargement of retroperitoneal venous collaterals [21] and preferential left renal, and consequently, left adrenal venous drainage via these collaterals either above or below the renal vein. This also has the potential to cause a slight elevation in IVC cortisol and/or aldosterone levels. The slight elevation of cortisol in the low IVC was not observed in all patients, which is likely to reflect variability in accessory adrenal venous drainage.

There was no significant difference between the IVC and REIV aldosterone levels, and these were statistically equivalent. Interestingly, low (< 100 pmol/l) and undetectable (< 60 pmol/l) IVC/REIV aldosterone levels were recorded in 10% and 7% of cases respectively. These levels were comparable in both IVC and REIV samples. This was despite a firm biochemical diagnosis of primary aldosteronism, appropriate cessation of interfering antihypertensive medication and normokalaemia. Similarly, low aldosterone levels (< 100 pmol/l) were also found by Kline et al. [22] who carried out AVS in sedated patients and found that 10% exhibited IVC aldosterone levels less than 100 pmol/l [22]. Recent studies have shown fluctuations in plasma aldosterone concentrations in patients with confirmed primary aldosteronism with lower levels of aldosterone on the day of AVS compared to workup values [5–7]. The drop in aldosterone levels on the day of AVS suggests that posture plays an important role in the observed variability as patients lie supine for much of the time prior to the AVS and during the procedure itself. As AVS cannot easily be performed in a seated position, the postural changes may be mitigated by minimising the length of time spent in the supine position prior to the procedure [5–7]. ACTH stimulation may increase aldosterone levels, although we do not use this as it results in some cases of unilateral disease being mislabelled as bilateral, resulting in patients who would benefit from surgery being denied it [12, 17, 23].

A low IVC sample inferior to the level of the renal veins is accepted as a surrogate for peripheral blood for the measurement of non-adrenal cortisol and aldosterone [24]. Peripheral veins can also be used as an alternative [25], with the femoral or iliac veins also being accepted [26]. In our institution, cortisol levels from the IVC have been utilised routinely for the SI calculation. Published studies using iliac venous samples, as a surrogate for a peripheral one, are limited [27, 28]. Whilst the small difference in cortisol levels between the IVC and REIV samples had no impact on the outcomes, the use of a more peripheral sample (e.g. REIV) may minimise the risk of any adrenal cortisol or aldosterone contaminating the IVC sample.

Some of the limitations of the study include its retrospective nature as well as all the procedures being performed by a single operator, which may make the results less generalisable.

## Conclusion

This study demonstrated that IVC cortisol and aldosterone levels were equivalent to those from the REIV. This translated into no discordance in selectivity or lateralisation. IVC and REIV samples may, therefore, be used interchangeably during AVS.
